# Antioxidant and Enzyme Inhibitory Potential of *Streptomyces* sp. G-18 Grown in Various Media

**DOI:** 10.1155/2023/6439466

**Published:** 2023-08-07

**Authors:** G. C. Ashok, Suman Prakash Pradhan, Krishna Kumar Karki, Aakriti Khadka, Aishwarya Bhandari, Bishnu Prasad Pandey

**Affiliations:** ^1^Department of Chemical Science and Engineering, Kathmandu University, Dhulikhel, Nepal; ^2^Aquatic Ecology Centre, Kathmandu University, Dhulikhel, Nepal

## Abstract

*Streptomyces* are bacteria well known for producing bioactive secondary metabolites which are commonly found in diverse habitats. The biosynthesis of metabolites from *Streptomyces* is influenced by various factors such as the growth medium, environmental conditions, and gene regulation. This study aimed to investigate the influence of different growth media on biomass production and the antioxidant and enzyme inhibitory potential of a crude extract obtained from *Streptomyces* sp. G-18 isolated from high altitudinal soil of Nepal. The highest dry weight growth was observed in R2YE medium (184 mg/L), followed by R5 (144 mg/L), YEME (38 mg/L), and R5M media (30 mg/L). The crude extract showed notable antioxidant activities against free radicals. The highest alpha-amylase inhibition was observed in the R2YE medium, and worthy lipase and tyrosinase inhibition was observed in the YEME medium. However, only the R2YE medium exhibited inhibitory potential against elastase and acetylcholinesterase, while crude extracts from R5, YEME, and R5 modified did not show any such activity. Overall, our findings suggest that the production of bioactive secondary metabolites in *Streptomyces* sp. G-18 was significantly influenced by the growth medium. This strain may be a promising source of enzyme inhibitors with potential applications in the pharmaceutical and cosmetic industries.

## 1. Introduction

*Streptomyces*, a Gram-positive bacterial genus in the family Streptomycetaceae and order Actinomycetales, has displayed a wide range of biochemical properties [1]. *Streptomyces* is distinguished from other bacteria in that it does not develop as a typical bacterial bacillary coccoid form but rather in a filamentous or mycelial form. Actinomycetes are unique in that they can grow on a wide variety of substrates present in the soil, including plant polymers such as chitin, cellulose, and hemicellulose, as well as animal products such as difficult-to-degrade insects [2]. They are unusual in the bacterial community due to their ability to survive in extreme environmental conditions of high pH, high temperature, and water stress [3]. They can be found in practically any environment, from the deep sea to the high mountains [4]. *Streptomyces*' most fascinating characteristic is its capacity to synthesize bioactive secondary metabolites that have a wide range of biochemical properties such as antifungals, antivirals, antitumoral, antihypertensives, immunosuppressants, and antibiotics [5]. *Streptomyces* are thought to be responsible for roughly one-third of the thousands of naturally occurring antibiotics [6]. Many secondary metabolites, including antibiotics, are produced in tandem with morphological distinction [7]. *Streptomyces'* secondary metabolites hinder a variety of biological processes; *Streptomyces* strains outperform other actinomycetes strains in terms of their capacity to generate a high number and variety of bioactive secondary metabolites. These secondary metabolites likely allow *Streptomyces* species to compete with other microbes, even those of the same genera [8].

*Streptomyces* has historically played a significant role in the identification of significant bioactive secondary metabolites, such as antibiotics, immunosuppressive medications, anticancer medicines, and other physiologically active chemicals [9]. Unfortunately, throughout the past few decades, comparable and well-known chemicals from terrestrial *Streptomyces* have continued to be discovered. In order to find novel bioactive compounds, it is advantageous to look for promising microorganisms in unknown or underexploited natural habitats with different isolation processes [10]. Given this, a lot of effort has been paid in recent years to the isolation of possible *Streptomyces* species in habitats with more severe settings, such as deep sea, desert, arctic, and volcanic environments. Several metabolites from *Streptomyces* are found to be effective in the formulation of antibiotics and several other medicines. Among the other uses, these metabolites are found effective in biochemical activities. Several *Streptomyces* have shown enzyme production activities (amylase, lipase, catalase, cellulose, protease, and asparaginase) that are of industrial importance [11]. These enzymes are being used in pharmaceuticals, laundry, cosmetics, paper, textile, fermentation processes, and wastewater treatment industries [12, 13]. In addition to this, several metabolites from *Streptomyces* are investigated for their potential antioxidant, anti-inflammatory, anticancer, antimicrobial, and several enzyme inhibitory potentials as well [14].

Inhibition of important enzymes is an effective strategy for the treatment and management of various diseases including Alzheimer's disease (AD) [15], diabetes mellitus (DM) [16], and dermatological disorders [17] in humans. Cholinesterase inhibitors, for example, raise acetylcholine levels in the brain, enhancing cognitive function in Alzheimer's patients [18]. Inhibitors of carbohydrate-digesting enzymes (*alpha*-amylase and *alpha*-glucosidase) help manage blood glucose levels in diabetic patients [19]. Similarly, inhibition of elastase and tyrosinase, which are involved in skin aging and pigmentation, are critical for cosmetic improvement [20, 21]. As a result, numerous synthetic inhibitors are utilized in clinical trials, but their efficacy is limited and they have some negative effects [22, 23]. Continuing efforts are being made to screen and create novel inhibitors from natural sources that are both effective and have fewer negative effects. In this study, we evaluated the antioxidant and enzymes inhibition potential of extract from *Streptomyces* sp. (G-18) grown and extracted on four different growth media.

## 2. Materials and Methods

### 2.1. Soil Collection and Species Isolation

To isolate *Streptomyces* species, soil samples were obtained from Gosaikunda, Langtang National Park, Rasuwa, Nepal (28.0820°N, 85.4150°E). *Streptomyces* species were isolated using the serial dilution technique in an ISP2 medium using a sterile spreader as described previously [13].

### 2.2. Chemical and Reagents

All the chemicals and reagent used in this work were of laboratory and analytical grade. Diphenyl-1-picrylhydrazyl (DPPH), 2, 2′-azino-bis (3-ethylbenzothiazoline-6-sulfonic acid) diammonium salt (ABTS), 3,5-dinitrosalicylic acid (DNSA), *α-*amylase, acarbose, 4-nitrophenyl butyrate (p-NPB), lipase, orlistat, l-3,4-dihydroxyphenylalanine (L-DOPA), mushroom tyrosinase, kojic acid, N-succinyl-Ala-Ala-p-nitroanilide (AAAPVN), porcine pancreatic elastase, quercetin, acetylcholinesterase (AChE), butyrylcholinesterase (BChE), acetylthiocholine iodide, butyrylcholine iodide, 5,5′-dithiobis-(2-nitrobenzoic acid) (DNTB), and galantamine were purchased from Sigma-Aldrich, USA.

### 2.3. Media Preparation and Bacterial Growth Optimization

Isolates of *Streptomyces* sp. (G-18) were grown in four different media [24]. The first is R2YE medium, which includes sucrose (103 g), glucose (10 g), 5 mL yeast extract (10%), MgCl_2_ (10.12 g), K_2_SO_4_ (0.25 g), and Difco Casamino acids (0.1 g) in 800 mL volume with distilled water. Then, 80 mL of the resulting solution was placed in a 250 mL Erlenmeyer flask and 1 mL KH_2_PO_4_ (0.5%), 8 mL CaCl_2_.2H_2_O (3.68%), 1.5 mL L-proline (20%), 10 mL TES buffer (5.73%; pH 7.2), 0.5 mL NaOH (1 N), 0.2 mL of trace element (ZnCl_2_ (80 mg), 400 mg FeCl_3_.6H_2_O), 40 mg each of CaCl_2_.2H_2_O, MnCl_2_.4H_2_O, NaB_4_O_7_.10H_2_O, and (NH_4_)_6_Mo_7_O_24_.4H_2_O were added. Yeast extract malt extract (YEME) medium contains yeast extract (3 g), peptone (5 g), malt extract (3 g), glucose (10 g), sucrose (340 g), and 2 mL MgCl_2_.6H_2_O (5 mM). The final volume was adjusted to 1000 mL with the addition of distilled water. The R5 medium includes sucrose (103 g), glucose (10 g), MgCl_2_.6H_2_O (10.12 g), K_2_SO_4_ (0.25 g), yeast extract (5 g), Casamino acids (0.1 g), TES buffer (5.73 g), 0.2 mL of trace element (ZnCl_2_ (80 mg), 400 mg FeCl_3_.6H_2_O), and 40 mg each of CaCl_2_.2H_2_O, MnCl_2_.4H_2_O, NaB_4_O_7_.10H_2_O, and (NH_4_)_6_Mo_7_O_24_.4H_2_O. The final volume was adjusted to 1000 mL with distilled water. To 100 mL of this solution in a 250 mL of Erelnmeyer flask, 1 mL KH_2_PO_4_ (0.5%), 0.4 mL CaCl_2_.2H_2_O (5 M), 1.5 mL L-proline (20%), and 0.7 mL NaOH (1 N) was added. The R5 modified (R5M) medium contained glucose (10 g), MgCl_2_.6H_2_O (10.12 g), K_2_SO_4_ (0.25 g), yeast extract (5 g), CaCO_3_ (2 g), Casamino acids (0.1 g), 2 mL of trace element (ZnCl_2_ (80 mg), FeCl_3_.6H_2_O (400 mg)), CaCl_2_.2H_2_O (20 mg), MnCl_2_.4H_2_O (20 mg), NaB_4_O_7_.10H_2_O (20 mg), and (NH_4_)_6_Mo_7_O_24_.4H_2_O (20 mg). The final volume was adjusted to1000 mL with the distilled water.

### 2.4. Extract Preparation and Growth Analysis

The growth of *Streptomyces* sp. (G-18) in different media was evaluated in 24 hrs intervals for a total 168 hrs. In brief, from each of the media, 1 mL of extracts was taken and dried to determine dry weight of the bacterial growth and repeated for seven days. Growth curve analyses were carried out by plotting the bacterial growth curve [25]. Furthermore, after 168 hrs of growth, metabolites were extracted using ethyl acetate as a solvent, dried using a vacuum evaporator, and used for antioxidant and enzyme inhibition assays.

### 2.5. Antioxidant Assays

Antioxidant activity of extracted metabolites was determined by following standard for 2, 2-diphenyl-1-picrylhydrazyl (DPPH) and 2, 2′-azino-bis (3-ethylbenzothiazoline-6-sulfonic acid) diammonium salt (ABTS) radical scavenging assays [19]. To measure the DPPH free radical scavenging, bacterial extracts at various concentrations (10, 20, 40, 60, 80, and 100 *μ*g/mL) were mixed with a 0.1 mM DPPH solution in a ratio of 1 : 3 and incubated for 30 min at dark. The absorbance was measured using a UV-visible spectrophotometer at 517 nm (Shimadzu UV-1800). For the ABTS assay, a sample of 1 mL of bacterial extracts at various concentrations (10, 20, 40, 60, 80, and 100 *μ*g/mL) was combined with 3 mL of ABTS working solution (mixture of ABTS and sodium potassium tartrate) and allowed to incubate in darkness for 10 min. The decrease in absorbance at the wavelength of 720 nm was measured using a UV-visible spectrophotometer. Ascorbic acid was used as a reference for both DPPH and ABTS. Moreover, methanolic DPPH was used as a control for the DPPH assay, and methanolic ABTS was used as a control for the ABTS assay.

### 2.6. *Alpha*-Amylase Assay

The 3,5-dinitrosalicylic acid (DNSA) method was used to determine *α-*amylase inhibition activity [19] in which 200 *μ*L each of extracts (20–360 *μ*g/mL) and 3 unit/mL *α-*amylase (Sigma-Aldrich, USA) were mixed and incubated for 15 min at 37°C. An additional 5 min of incubation at 37°C was carried out after the addition of 200 *μ*L of 1% starch solution. Then, a 200 *μ*L of DNSA solution was added to it and the whole reaction mixture was heated for 10 min at 95°C. The final volume was adjusted to 5 mL with the addition of distilled water. The absorbance was measured at *λ* = 540 nm in a UV-visible spectrophotometer. The results were compared with the activities of acarbose and 1% dimethyl sulfoxide (DMSO) was used as a positive control.

### 2.7. Lipase Inhibition Assay

The standard method of measuring the lipase activity was adapted by using 4-nitrophenyl butyrate (p-NPB) as a substrate [26]. In brief, 80 *μ*L of extracts (10–160 *μ*g/mL) were mixed with 40 *μ*L of substrate solution (10 mM of p-NPB in ethanol) and 80 *μ*L of the lipase enzyme (2.5 mg/mL in 0.1 M phosphate buffer, pH 8.0). After incubation (20 min; 37°C), absorbance was observed at 405 nm using a 96-microplate reader (BioTek, EPOCH). The results were compared with the activities of orlistat and 1% DMSO was used as a positive control.

### 2.8. Tyrosinase Inhibition Assay

The l-3,4-dihydroxyphenylalanine (L-DOPA) substrate was used to determine tyrosinase inhibition activity [27]. In brief, 40 *μ*L extracts (10–160 *μ*g/mL) in potassium phosphate buffer (0.05 M, pH 6.5) were mixed with 15 *μ*L of mushroom tyrosinase enzyme (500 U/mL) and incubated at 27°C for 10 min. Then, by adding 20 *μ*L of L-DOPA (5 mM), the reaction volume was maintained to 200 *μ*L by the addition of potassium phosphate buffer and incubated for an additional 30 min. Absorbance was measured at 492 nm in a 96-microplate reader. The results were compared with the activities of kojic acid.

### 2.9. Elastase Inhibition Assay

The N-succinyl-Ala-Ala-p-nitroanilide (AAAPVN) substrate was used to assess the elastase inhibitory activity, following the Ellman method with minor modifications [28]. In brief, extracts of (10–160 *μ*g/mL) were mixed with porcine pancreatic elastase (0.05 U/mL) and 50 g/mL AAAPVN and incubated for 30 min at 25°C. With the addition of 0.2 M Tris-HCl buffer (pH 8.0), the final reaction volume was maintained at 200 *μ*L. In a 96-microplate reader, absorbance was measured at 410 nm. As a positive control, 1% DMSO was used, and the results were compared with the activities of quercetin.

### 2.10. Cholinesterase Inhibition Assay

The analysis of inhibition of acetylcholinesterase (AChE) and butyrylcholinesterase (BChE) was conducted [29]. To begin, 0.05 U/mL AChE or 0.5 U/mL BChE was combined with extracts (10–160 *μ*g/mL) and incubated at 25°C for 15 min. The reaction mixture was then supplemented with 1 mM acetylthiocholine iodide or 1.5 mM butyrylcholine iodide and 0.5 mM of 5,5′-dithiobis-(2-nitrobenzoic acid) (DNTB). By adding 0.1 M sodium phosphate buffer (pH 8.0), the total reaction volume was maintained at 200 *μ*L. The absorbance was determined at 412 nm using a 96-microplate reader. 1% DMSO was used as positive control and results were compared with the activities of galantamine.

### 2.11. Statistical Analysis

The experiments were performed in triplicate for each analysis and the results are presented as mean ± standard deviation (mean ± SD). The percentage of radicals scavenging and enzymes inhibition was determined by calculating the difference between the values of positive control and examined extracts as shown in the following formula [29]. Moreover, the inhibitory concentration at which absorbance is 50% (IC_50_) values were calculated by linear regression analysis of the percentage of radicals scavenging and percentage of enzymes inhibition. The significant difference in the mean value of examined extracts was analyzed by the one-way analysis of variance (one-way ANOVA), post hoc Tukey, and multiple comparison test at 95% confidence level (*p* < 0.05) by using SPSS version 26.

(1) $$\begin{array}{c} \%\;Inhibition=\frac{Absorbance\;of\;Control-Absorbance\;of\;Test*100}{Absorbance\;of\;Control}\text{.} \end{array}$$

## 3. Results and Discussion

### 3.1. Growth Pattern

**Figure 1:**
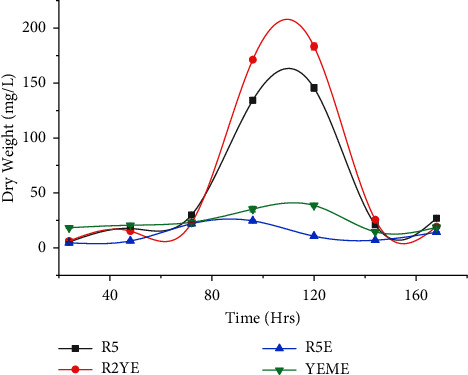
Growth curve of *Streptomyces sp.* (G-18) grown in R2YE, YEME, R5, and R5M medium. The experiments were conducted in triplicate, and the results were presented as the mean value accompanied by the ±standard deviation (mean ± SD).

During the growth phase, *Streptomyces* produces secondary metabolites, which are substances that are not strictly necessary for the organism to grow or reproduce but can provide it with a competitive edge [9]. These metabolites promote vegetative bacterial cells by protecting metals like iron (siderophores), protecting them from UV radiation (through coloring), thwarting competitors (antibiotics), and encouraging communication with other species [30]. This genetic diversity is made possible by the unusually large genome of *Streptomyces*, which may be triple the size of certain other bacterial genomes [31]. Past efforts have been carried out to screen and identify the potential antibiotics-producing *Streptomyces* species from diverse geographical locations. We have previously identified *Streptomyces* species isolated from the high altitude of Nepal as a potential source of antimicrobial compounds [13]. In this study, four different media were used (R2YE, YEME, R5, and R5M) to grow *Streptomyces* sp. (G-18) and were evaluated for their antioxidant and enzyme inhibition potential. We observed the Monod growth pattern [32] with a lag phase varying from 48 to 72 hours, and *Streptomyces* sp. (G-18) grew exponentially until 96 hours. The stationary phase lasted from 96 to 120 hrs, and the death phase began around 144 hrs (Figure 1). The maximum dry weight increase was obtained on R2YE media (184 mg/L), followed by R5 media (144 mg/L), YEME media (38 mg/L), and R5M media (30 mg/L).

### 3.2. Antioxidant Activities

**Figure 2:**
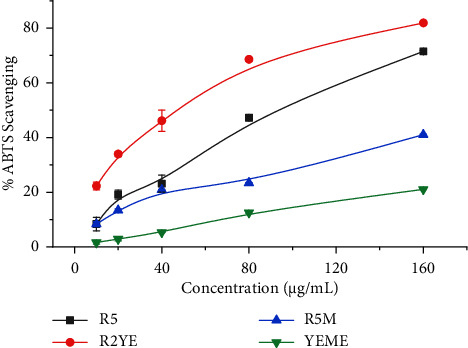
Percentage of ABTS scavenging by *Streptomyces sp.* (G-18) extracts. The experiments were conducted in triplicate, and the results were presented as the mean value accompanied by the ±standard deviation (mean ± SD).

**Figure 3:**
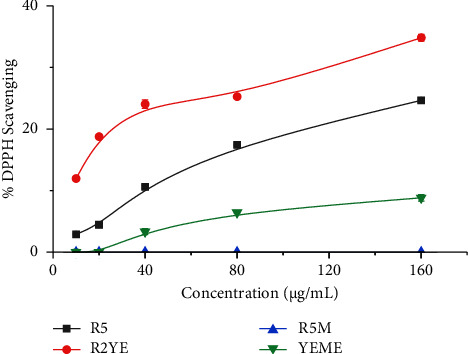
Percentage of DPPH scavenging by *Streptomyces sp.* (G-18) extracts. The experiments were conducted in triplicate, and the results were presented as the mean value accompanied by the ±standard deviation (mean ± SD).

Free radicals, especially reactive oxygen species (ROS) cause cellular damage. An abnormal buildup of free radicals is likely to arise from both endogenous and exogenous metabolism. To counteract the effects of these free radicals, antioxidants are produced in humans and other organisms [33]. Antioxidant supplementation is required to reduce high levels of ROS production and prevent harmful effects on cells, tissues, and organs that are linked to various ailments [34]. Among the analyzed extracts, our results revealed that *Streptomyces* sp. (G-18) grown in the R2YE medium had the highest ABTS scavenging capabilities with 82% inhibition (IC_50_ = 61 ± 1.5 *μ*g/mL), and YEME extracts showed the lowest ABTS scavenging potential with 21% inhibition (IC_50_ = 370 ± 10.0 *μ*g/mL) (Table 1 and Figure 2). The most DPPH radical scavenging was also observed in R2YE medium extracts with 35% inhibition (IC_50_ = 240 ± 7.0 *μ*g/mL). In contrast, the R5M medium extract did not show inhibition against DPPH (Table 1 and Figure 3). Our results fit with the findings of Kemung et al. who reported *Streptomyces* sp. MUSC 14 isolated from mangrove forest soil in Malaysia shows significant antioxidant activities when analyzed using ABTS and DPPH [35]. Similarly, *Streptomyces* sp. MUM212 grown in ISP2 media extract demonstrated significant antioxidant activity through DPPH, ABTS, superoxide radical scavenging, and also metal-chelating activity of 22%, 62%, 37%, and 42% at 4 mg/mL, respectively [36]. Based on our findings along with previous findings, we postulated that medium composition plays an important role in the expression of several enzymes and biomolecules that govern the activities of *Streptomyces* extracts.

### 3.3. *Alpha*-Amylase and Lipase Inhibition

**Figure 4:**
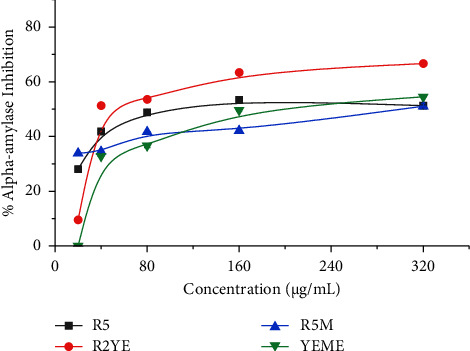
Percentage of alpha-amylase inhibition by *Streptomyces sp.* (G-18) extracts. The experiments were conducted in triplicate, and the results were presented as the mean value accompanied by the ±standard deviation (mean ± SD).

**Figure 5:**
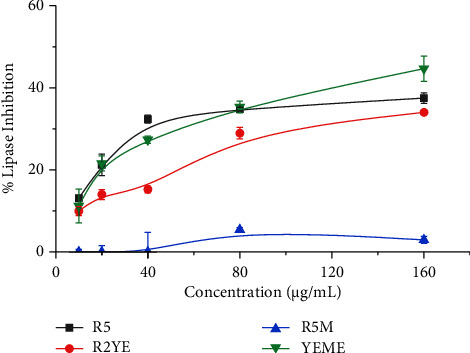
Percentage of lipase inhibition by *Streptomyces sp.* (G-18) extracts. The experiments were conducted in triplicate, and the results were presented as the mean value accompanied by the ±standard deviation (mean ± SD).

Of the analyzed enzymes, *alpha*-amylase breaks down the glycosidic linkage, releasing glucose. Hence, inhibitors of *alpha*-amylase help reduce glucose concentration through carbohydrate metabolism. Furthermore, obesity is linked with the abnormal breakdown and deposition of fat in the body, which is associated with several health issues such as heart disease, cancer, osteoarthritis, hypertension, and diabetes [37]. Hence, the pharmaceutical and food industries are looking for natural inhibitors. Our results with the extracts of *Streptomyces* sp. G-18 grown in four different media show potential inhibitors of enzymes, such as lipase and *alpha*-amylase, except for the R5M medium. Among the four media, *Streptomyces* sp. G-18 grown in the R2YE medium revealed the highest *alpha*-amylase inhibition with 66% inhibition (IC_50_ = 130 ± 0.5 *μ*g/mL). However, R5M medium extract showed the least inhibition against *alpha-*amylase with 51% inhibition (IC_50_ = 280 ± 1.0 *μ*g/mL) (Table 2 and Figure 4). YEME exhibited the highest lipase inhibition at 45% inhibition (IC_50_ = 157 ± 6.0 *μ*g/mL) followed by R5 and R2YE. R5M media extract revealed no inhibition of lipase (Table 2 and Figure 5). A similar work carried out by Siddharth and Vittal provided evidence of *Streptomyces* sp. S2A inhibits *alpha*-amylase and *alpha*-glucosidase with IC_50_ values of 21 *μ*g/mL and 20 *μ*g/mL, respectively [38]. Statistical analysis (One-way ANOVA) revealed a significant difference in the results of the inhibition measurements of each extract against *alpha*-amylase and lipase enzymes (*p* < 0.05) (Table 2). In previous studies, Cyclipostins produced by *Streptomyces* sp. DSM 13381 and lipstatin produced by *Streptomyces toxytricini* showed inhibition against lipase enzymes [39, 40]. Our work is the first to evaluate the lipase inhibitory activity of *Streptomyces* G-18 extracts grown in different media. The extracting medium and concentration dependent inhibition pattern for enzymes indicates the importance of growth medium selection for the optimization of biochemical activities.

### 3.4. Tyrosinase and Elastase Inhibition

Elastase is a protease that belongs to the chymotrypsin family and is responsible for the degradation of elastin [41], a protein that is present in the extracellular matrix (ECM). It is crucial for preventing skin aging (natural as well as premature) caused by intrinsic and extrinsic factors [42]. The search for potent elastase inhibitors from natural sources is an alternative to commercially available chemical-based skin aging suppressors. Among the analyzed four extracts, only the R2YE medium revealed 54% elastase inhibition (IC_50_ = 140 ± 7.0 *μ*g/mL) (Table 2). A previous study with the extract from *Streptomyces* KM-2753 showed noteworthy elastase inhibition [43].

**Figure 6:**
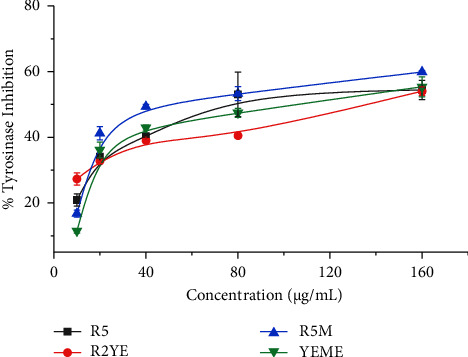
Percentage of tyrosinase inhibition by *Streptomyces sp.* (G-18) extracts. The experiments were conducted in triplicate, and the results were presented as the mean value accompanied by the ±standard deviation (mean ± SD).

Tyrosinase is a multicopper enzyme that is involved in both melanogenesis and enzymatic browning [44]. As a result, tyrosinase inhibitors may be appealing as depigmentation agents in the cosmetics and pharmaceutical sectors, as well as in food and agricultural industries as antibrowning agents. To date, numerous natural, semisynthetic, and synthetic inhibitors have been produced for this purpose using various screening approaches [44]. Our study revealed that all the analyzed crude extracts to have tyrosinase inhibitory activity. R5M medium extract had the highest tyrosinase inhibitory activity, with IC_50_ values of 78 ± 2.0 *μ*g/mL (60% inhibition) followed by YEME, R5, and R2YE medium extracts (Table 2 and Figure 6). A significant difference in inhibition activity of our analyzed extracts and those reported previously was observed for both elastase and tyrosinase inhibition (*p* < 0.05). Previous work reported several *Streptomyces* extracts to have tyrosinase inhibition capabilities. For example, *Streptomyces hiroshimensis* TI-C3 was isolated from soil and showed antityrosinase activity (498 U/mL) with improved activity (905 U/mL) when glucose and malt extract were used as the only carbon and nitrogen sources, respectively [45]. *Streptomyces roseolilacinus* NBRC 12815 developed two antityrosinase compounds, 12815 A (IC_50_ = 9 M) and B (IC_50_ = 1086 M), which were tested against mushroom and mammalian tyrosinases [46]. These results support a potential application of *Streptomyces* extracts in the cosmetic industry with our emphasis that extraction medium and processes should be taken into consideration.

### 3.5. Cholinesterase Inhibition Activities

Alzheimer's disease (AD) is a neurological syndrome characterized by abnormal behavior and intellectual decline [47]. The deficiency of acetylcholine and butyrylcholine in the human brain is important for the proper functioning of neuromediators [48]. Inhibiting the AChE and BChE enzymes, which are responsible for the hydrolysis of AChE and BChE neurotransmitters, has therefore become a therapeutic option for AD [49]. Alkaloids are the greatest source of AChE and BChE inhibitors as therapeutic agents against AD [50]. Among our analyzed crude extracts, YEME and R5M medium extracts did not reveal any inhibitory activity toward cholinesterases (Table 2). Moreover, only the R2YE medium extract showed AChE inhibition (37 ± 0.3%; IC_50_ = 137 ± 1.0 *μ*g/mL), and only the R5 medium extract revealed 33 ± 0.9% inhibition against BChE enzyme (IC_50_ = 200 ± 4.0 *μ*g/mL). A recent study showed that Geranylphenazinediol produced by *Streptomyces* sp. LB173 inhibited AChE in the low micromolar range [51]. Moreover, marine-derived *Streptomyces* sp. UTMC 1334 revealed potent AChE inhibition [52]. Also, chromenone derivatives from marine-derived *Streptomyces* sp. CNQ-031 showed effective cholinesterase inhibition [53]. In our study, a significant difference between AChE and BChE inhibition by our extracts and reference used was observed (*p* < 0.05) in (Table 2). The selectivity of *Streptomyces* strain in producing secondary metabolites that act against these enzymes might be the reason for medium discerning inhibition potential.

## 4. Conclusion

In conclusion, *Streptomyces* sp. G-18 is a potential source of enzymes inhibitors for which metabolites production and regulation are largely dependent on the growth medium and culture conditions. We observed R2YE and R5 medium as the best-suited medium for optimal growth and are associated with the highest biological activities. Our results open up the possibility to find other major secondary metabolites produced by *Streptomyces* sp. G-18 for pharmaceuticals and cosmetic industries.

## Figures and Tables

**Table 1:** Radical scavenging activities of *Streptomyces sp.* (G-18) extracts. The experiments were conducted in triplicate, and the results were presented as the mean value accompanied by the ±standard deviation (mean ± SD).

Samples	IC_50_ (*μ*g/mL)	
	ABTS	DPPH
R5M	199 ± 1.5^d^	NI
R5	100 ± 1.8^c^	309 ± 2.7^c^
YEME	371 ± 10^e^	550 ± 16^d^
R2YE	61 ± 1.6^b^	244 ± 7.20^b^
Gallic acid	2 ± 0.05^a^	5 ± 0.1^a^

NI; no inhibition. Different letters in the superscript of same column represents the significant difference in mean values, one-way ANOVA, post hoc Tukey, multiple comparison test (*p* < 0.05).

**Table 2:** Enzyme inhibition activities of *Streptomyces sp.* (G-18) extracts. The experiments were conducted in triplicate, and the results were presented as the mean value accompanied by the ±standard deviation (mean ± SD).

Samples	IC_50_ (*μ*g/mL)					
	Alpha-amylase	Lipase	Tyrosinase	Elastase	AChE	BChE
R5M	280 ± 0.8^e^	NI	78 ± 1.8^b^	NI	NI	NI
R5	167 ± 3.0^c^	166 ± 5.4^b^	96 ± 8.0^c^	NI	NI	200 ± 4.0^b^
YEME	178 ± 0.3^d^	157 ± 5.8^a^	94 ± 2.0^c^	NI	NI	NI
R2YE	128 ± 0.5^b^	225 ± 12^c^	128 ± 5.1^d^	141 ± 7.1^b^	137 ± 1.0^b^	NI
Acarbose	20 ± 0.1^a^	—	—	—	—	—
Orlistat	—	432 ± 14^d^	—	—	—	—
Kojic acid	—	—	18 ± 0.1^a^	—	—	—
Quercetin	—	—	—	101 ± 0.2^a^	—	—
Galantamine	—	—	—	—	1 ± 0.02^a^	26 ± 1.4^a^

NI; no inhibition. Different letters in the superscript of same column represents the significant difference in mean values, one-way ANOVA, post hoc Tukey, multiple comparison test (*p* < 0.05).

## Data Availability

The data used to support the findings of this study are available from the corresponding author upon reasonable request.
